# Editorial: Viruses, Genetic Exchange, and the Tree of Life

**DOI:** 10.3389/fmicb.2019.02782

**Published:** 2019-11-29

**Authors:** Arshan Nasir, Gustavo Caetano-Anollés, Jean-Michel Claverie

**Affiliations:** ^1^Department of Biosciences, COMSATS University Islamabad, Islamabad, Pakistan; ^2^Theoretical Biology and Biophysics, Los Alamos National Laboratory, Los Alamos, NM, United States; ^3^Evolutionary Bioinformatics Laboratory Department of Crop Sciences, University of Illinois at Urbana-Champaign, Urbana, IL, United States; ^4^Aix Marseille University, CNRS, IGS, Structural and Genomic Information Laboratory (UMR7256), Mediterranean Institute of Microbiology (FR3479), Marseille, France

**Keywords:** viruses, tree of life, evolution, horizontal gene transfer, ORFans, origin of nucleus

We live in exciting times for microbiology research. The significant technological and scientific advancements in the past decades have now enabled scientists to pursue discovery and description of novel microbial and viral linages from previously uncharted Earth habitats. Two such discoveries are particularly exciting and noteworthy in this regard. First, the discovery of the first “giant virus,” *Acanthamoeba polyphaga mimivirus*, in 2003, and several others thereafter, posed intriguing questions regarding virus origins, evolution, classification, and their place in the “tree of life.” Second, the discoveries of “Lokiarchaeota” and several other closely-related archaeal members that encode several eukaryote-specific proteins challenged the three-domain canonical structure of the tree of life. These discoveries have reopened debates on central questions in evolutionary biology research such as the origin of viruses, the origin of eukaryotes, evolutionary relationship between Archaea and Eukarya, and the structure and topology of the tree of life. In this Research Topic, we received a broad range of contributions addressing these and other related questions.

Moelling and Broecker discussed the “virus first” model for the evolution of life on Earth. They provided several examples of virus diversity and abundance in a range of Earth environments and in the mammalian genomes. According to their view, ribozymes and viroids could have started early evolution albeit they also acknowledged competing alternatives such as the “proteins first” and the “metabolism first” scenarios of origins of life. In a separate contribution from the same authors, they examined the possibility that complex antiviral defense strategies and immune systems in cellular organisms could have evolved from viruses and transposable elements. The authors thus highlighted the crucial roles viruses could have played during the evolution of cells.

Ramisetty and Sudhakari discussed why the selection of “grounded prophages” may be favored by bacterial cells. Grounded prophages are unable to excise from bacterial genomes due to mutations in either recombinase gene or genes encoding attachment sites. Lysogens with grounded prophages are protected from specific phage infections and the future activation of lytic cycles. These grounded prophages can also serve as hotspots or buffer zones where genes encoding antibiotic resistance and virulence may integrate. Their work thus highlights another important contribution of viruses to the evolution of cells that contradicts the view of viruses as mere cellular pathogens.

Legendre et al. highlighted an often-ignored feature of viral genomes, the existence of many protein-coding genes with no detectable homologs. These virus-encoded ORFans constitute the large majority of genes of giant viruses but are mostly ignored when discussing models of virus evolution, while their origin remains a mystery. The authors provided evidence that ORFans in pandoraviruses, the largest viruses known to date, can be unique even among closely related members of the same virus family. These ORFans likely originate from intergenic regions and suggest that the pandoravirus pan-genome is open. These findings indicate that the genomes of viruses are incredibly dynamic in their gene creation capabilities, a notion hardly acknowledged in standard virus evolutionary scenarios where all genes are bound to have an ancestor.

The presence of nucleus is a defining distinction between eukaryotes and prokaryotes. While nucleus-like compartmentation has been described or proposed in planctomycetes and jumbophage-infected bacteria, it is unclear how these compartmentations are linked to the origin of nucleus in eukaryotes. Hendrickson and Poole presented several analogies of nucleus-like compartmentation outside eukaryotes and discussed three possible explanations for the emergence of compartmentation in cells: physical protection, crosstalk avoidance, and non-adaptive origins.

Harrison et al. explored the diversity of ribonucleotide reductases (RNRs) from marine viroplankton, ancient enzymes that reduce ribonucleotides to deoxyribonucleotides and are prominent in viral genomes. They found cyanophage Class II RNR enzymes were misannotated and were actually part of the large oxygen-dependent Class I clade. Since the marine *Synechococcus* and *Prochlorococcus* hosts carry only Class II enzymes, the finding confirms that the viroplankton RNRs are not host derived nor dependent on the adenosylcobalamin (B12) cofactor of class II enzymes. This is an important clarification for connecting genomic information and phenotypic traits in the context of viral ecology.

Wang et al. analyzed the genomes of 21 Coxsackievirus A4 isolates from hand, foot and mouth disease cases in children from the Shandong province of China. Phylogenetic analysis of the VP1 gene, when benchmarked to a large Coxsackievirus collection, revealed that genotypes C, D1, and D2 identified in the early 2000s have been taken over by the D2 genotype in China. Calculation of substitution rates revealed ongoing virus evolution and several dynamic fluctuations in the history of the virus isolates. The study is important for the molecular epidemiological characterization of A4, which has been understudied because of the paucity of genomic data.

Flynn and Moreau engaged in a comparative host-centered exploration of endogenous viral elements (EVE) in ants. Using a comprehensive bioinformatic pipeline, they screened all 19 published ant genomes for EVEs and assessed their phylogenetic relationships to closely related exogenous viruses. EVEs similar to proteins from single stranded RNA viruses, viral glycoproteins, and retro-virus-derived proteins were widely present in ant genomes, suggesting tendencies to endogenize. EVEs therefore mimic the diversity of viral lineages.

Ongrádi et al. isolated an adenovirus from a domestic cat and found it was related to human adenovirus. Their careful molecular, biological, and phylogenetic characterizations highlight important information with the potential to re-define the adenovirus research field. It also prompts a broader analysis of adenoviruses of many different types. Further epidemiological and pathomechanistic studies can help understand the molecular mechanisms of virus host jumps, which could be engineered to combat both feline and human AIDS.

Flodman et al. performed an extensive analysis of interaction between commercially available restriction enzymes and bacteriophages containing modified nucleotides. They evaluated restriction resistance of phages M6, Vil, and phi W-14 against 200 commercially available enzymes. This work has enormous value for genetic engineering and understanding interaction of proteins with virus-modified DNA.

Sun et al. showed evidence that polysaccharides isolated from the leaves of the *Aloe vera* plant, known to mitigate virus infection, inhibit the replication of a H_1_N_1_ subtype of influenza virus at the time of virus adsorption. The anti-influenza activities were for the first time explored *in vitro* in cell cultures and in a mouse model. This study opens the door to the development of novel anti-influenza drugs.

Finally, Zhou et al. presented a novel mismatch-tolerant loop-mediated isothermal amplification (LAMP) method with an efficiency high enough to be applied to the detection of viruses. The methodology tolerates mismatches in primers and templates. They used this new methodology to detect variants of antigenically-distinct serotypes of the dengue virus.

The Research Topic is thus a collection of ideas ranging from our basic understanding of virus origins and evolution to new approaches of virus identification and treatment. We hope this resource will be a useful reference for future studies in basic and applied virology.

## Author Contributions

All authors listed have made a substantial, direct and intellectual contribution to the work, and approved it for publication.

### Conflict of Interest

The authors declare that the research was conducted in the absence of any commercial or financial relationships that could be construed as a potential conflict of interest.

